# Cancer as an Embryological Phenomenon and Its Developmental Pathways: A Hypothesis regarding the Contribution of the Noncanonical Wnt Pathway

**DOI:** 10.1155/2019/4714781

**Published:** 2019-03-03

**Authors:** Jaime Cofre, Kay Saalfeld, Eliana Abdelhay

**Affiliations:** ^1^Laboratório de Embriologia Molecular e Câncer, Universidade Federal de Santa Catarina, Sala 313b, 88040-900 Florianópolis, SC, Brazil; ^2^Laboratório de Filogenia Animal, Universidade Federal de Santa Catarina, Brazil; ^3^Divisão de Laboratórios do CEMO, Instituto Nacional do Câncer, Rio de Janeiro, Brazil

## Abstract

For gastrulation to occur in human embryos, a mechanism that simultaneously regulates many different processes, such as cell differentiation, proliferation, migration, and invasion, is required to consistently and effectively create a human being during embryonic morphogenesis. The striking similarities in the processes of cancer and gastrulation have prompted speculation regarding the developmental pathways involved in their regulation. One of the fundamental requirements for the developmental pathways in gastrulation and cancer is the ability to respond to environmental stimuli, and it has been proposed that the Kaiso and noncanonical Wnt pathways participate in the mechanisms regulating these developmental pathways. In particular, these pathways might also explain the notable differences in invasive capacity between cancers of endodermal and mesodermal origins and cancers of ectodermal origin. Nevertheless, the available information indicates that cancer is an abnormal state of adult human cells in which developmental pathways are reactivated in inappropriate temporal and spatial contexts.

## 1. Epigenetic Control Systems: The Developmental Pathways of Cancer

In the search for effective new cancer therapies, the embryo arises as a promising alternative for the identification of specific molecular targets within several embryonic developmental pathways (EDPs). Because the theoretical assumptions postulated by researchers are based on embryology [1] and included within the conceptual framework of epigenetics [this term encompasses two main aspects of the conceptual definition: changes in cellular composition (cellular differentiation) and changes in geometrical form (gastrulation) [2]], the demand for these “EDPs” should certainly be restricted to epigenetic molecular mechanisms within the embryo. Moreover, conceptual premises also highlight the embryological plasticity and canalization described by Waddington [2]. Additionally, based on the conceptual definition of epigenetics by Eva Jablonka at higher levels of biological organization, epigenetic mechanisms produce context-dependent, self-sustaining interactions between groups of cells that undergo physiological and morphological persistence, such as gastrulating cells [3]. The so-called morphological persistence must not be interpreted as a physical and concrete structure of the embryo that arises at a particular time and continues until the end of embryogenesis but rather as a morphological event that is temporally restricted and can produce a significant number of cells. Thus, these cells would truly be responsible for producing the deep structural changes necessary for final embryo consolidation.

An analysis of gastrulation (and possibly other embryonic stages) will likely reveal the origin of morphological persistence, with all the profound implications of such a process, at the cell and tissue level for cellular differentiation and determination as well as cancer, as will be discussed below. Thus, the epigenetic mechanisms that establish and maintain these cellular differences and organismal states, such as gastrulation, will be referenced here as epigenetic control mechanisms, the epigenetic regulatory machinery or simply epigenetic control systems [4]. Therefore, we speculate that an EDP must comprise the minimal conditions required to play a decisive role in regulating both embryogenesis and cancer by (1) participating in an epigenetic control system during gastrulation, (2) responding to external environmental stimuli, (3) functioning as a simultaneous regulator of various processes, such as cellular differentiation, proliferation, migration, and invasion, and (4) having a close relationship to adherens junctions and thereby creating a rich interface of epigenetic modulation, with some proper sense for “gastrulation” and “cancer”.

Now, we are going to describe a developmental pathway (among many others that may exist) that meets the minimal conditions for an EDP, described above, and included within the premises of our theoretical framework, and therefore, it could control both embryogenesis and cancer.

## 2. The Kaiso Pathway Meets the Minimal Conditions for the Developmental Pathways of Cancer

### 2.1. Kaiso as an Epigenetic Control System

Perhaps the best way to start a discussion of some developmental pathways of cancer in the framework of the present hypothesis is to consider methyl-CpG-binding domain proteins (MBD) that read and translate DNA-methylation marks and are thus critical mediators of several epigenetic processes [5, 6]. In particular, we focus on one nonclassical MBD protein called Kaiso, which contains a zinc-finger DNA-binding domain responsible for Kaiso-mediated transcriptional repression [7]. Kaiso and its partner, p120ctn, are similar to the *β*-catenin-T-cell factor (TCF)/lymphoid-enhancing factor (LEF) complex, but only Kaiso interacts with the epigenome during cancer development [8].

The Kaiso protein appears to be the only factor that showed bimodal features in its interaction with DNA: it is able to specifically interact with methylated regions called CpG islands and with the nonmethylated consensus sequence 5′-CTGCNA-3′ [9–11] (see the review by Fournier et al. [12]). The recognition of methylated DNA by Kaiso (through the methyl-CpG-binding domain) is essential for the epigenetic silencing of tumor-suppressor genes, a critical role that was previously characterized during colon cancer development [13]. In contrast, an important discovery that illuminates the function of methylation-mediated repression showed that Kaiso interacts with a corepressor complex containing histone deacetylase (HDAC) [14, 15]. HDAC catalyzes the deacetylation of histones, and these chemical modifications facilitate a more closed chromatin conformation and thereby reduce gene expression. Thus, the participation of Kaiso as a component of the human HDAC-containing corepressor complex might explain its extended role in genome-wide transcriptional repression, which is considered a very important process during the early embryogenesis of vertebrates [16].

### 2.2. Role in Embryogenesis during Gastrulation

To define the role of Kaiso in embryogenesis, we must first understand that the direct target gene of Kaiso in vertebrates is Wnt11, which belongs to the Wnt family of secreted signaling glycoproteins that exert their functions by activating noncanonical Wnt pathways. Noncanonical Wnt pathways are involved in the polarity and movement of cells via the epithelial-mesenchymal transition (EMT) observed during gastrulation [17, 18], and for this reason, Kaiso plays an essential role in controlling animal morphogenesis [19]. Consistent with these findings, Kaiso depletion in vertebrates produces severe alterations in gastrulation-related movements and consequently affects endoderm formation and neurulation. In humans, the Wnt11 promoter contains a conserved Kaiso-binding site (at -775), and experimental laboratory results have indicated cooperation between *β*-catenin/TCF and Kaiso/p120ctn in the negative regulation of Wnt11 transcription in chronic myeloid leukemia (CML) [20].

#### 2.2.1. Influence of Environmental Stimuli

The most intriguing aspect of the Kaiso protein is its intracellular localization. In cultured cell lines, Kaiso is almost exclusively located in the nucleus, where it functions as a transcriptional repressor [21, 22]. Surprisingly, several studies have revealed striking differences between the behaviors of different cell lines in monolayer cell culture systems, three-dimensional cell culture systems and even “in vivo” [23]. Although the nuclear localization of Kaiso has been observed in human tissues (both normal and tumor tissues), Kaiso is more frequently observed in the cytoplasm and is absent in some cell types. In contrast, the subcellular cytoplasmic localization of Kaiso is directly related to the poor prognosis of patients with non-small-cell lung cancer and CML [20, 24]. These results strongly indicate that the microenvironment has an unexpected influence on Kaiso expression and localization. Thus, knowledge of the complex regulatory dynamics that occur in response to the microenvironment during both embryogenesis and cancer progression is a fundamental requirement.

#### 2.2.2. Simultaneous Regulation of Different Processes during Gastrulation

We will now develop some ideas about additional requirements to enrich the conceptual basis of the hypothesis and continue discussing the minimal conditions, particularly those related to the simultaneous regulation of different processes that enable gastrulation, within the logic of the embryonic cancer model. We will briefly consider the participation of the Kaiso/p120ctn complex in four significant pathways that underlie the gastrulation process, all of which are intrinsically linked to the EMT. Therefore, we believe that an integrated epigenetic view of embryonic development might change the direction of the search for new cancer therapies by considering proteins that jointly regulate the processes involved in the EMT.

The Kaiso protein is a transcription factor with an broad-complex, tramtrack, and bric-a-brac proteins (BTB)/POX virus and zinc-finger (POZ) protein-protein interaction domain at the amino terminus and a zinc-finger DNA-binding domain at the carboxy terminus [21, 25]. The amino-terminal domain of Kaiso specifically interacts with its partner catenin p120 (p120ctn) [21]. A fascinating function of Kaiso is its ability to regulate TCF/LEF1 activity by modulating the formation of the HDAC1-*β*-catenin complex [26], and Kaiso and TCF/LEF1 appear to interact in the nucleus [27]. Thus, Kaiso/p120ctn might be required for morphogenetic processes in the embryo through direct modulation of any of the activities regulated by both the canonical Wnt pathway (and target genes of *β*-catenin) [15] and the noncanonical Wnt pathway (through the Wnt11 gene) [19]. This particular modulatory mechanism might have significant implications for the regulation of genes involved in cancer-associated processes.

**Figure 1:**
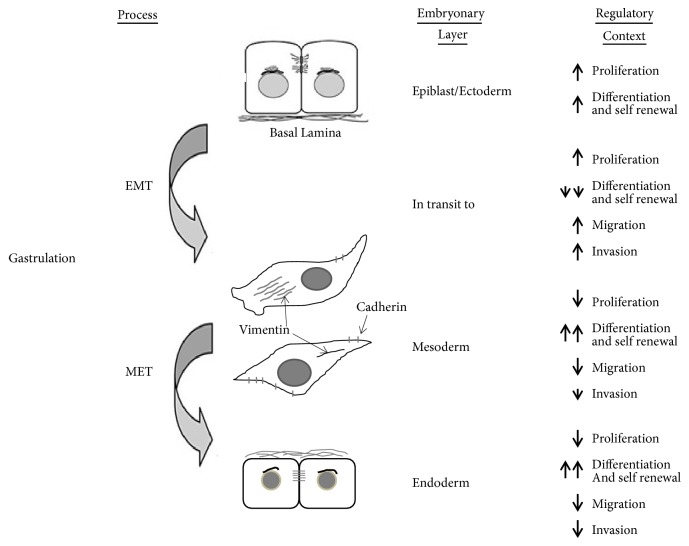
Differences established during gastrulation through the EMT and MET processes. At the end of gastrulation the distinct germ layers will be able to regulate and control, in a different way, the cellular processes such as proliferation, differentiation, migration, and invasion.

**Figure 2:**
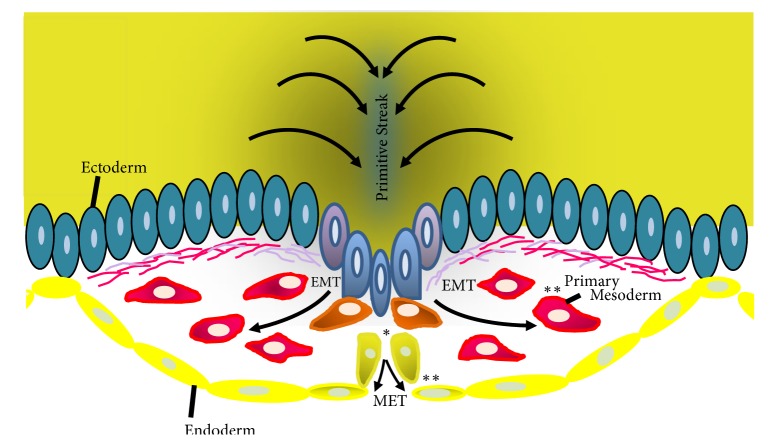
Human gastrulation and Kaiso. During gastrulation, ectodermal cells at the primitive streak undergo epithelial-mesenchymal transition (EMT) as a result of signals produced by the Spemann organizer or internal determinants. Also during this process the epiblast cells, in response to these signals, translocate Kaiso to the cytoplasmic compartment (*∗*) and thus these cells ingress through the primitive streak and migrate into the underlying tissue. In a second critical moment, the delaminating cells translocate Kaiso to the nuclear compartment (*∗∗*) and will form the primary mesoderm or will undergo mesenchymal-epithelial transition (MET) establishing the endoderm. Adapted from [1].

*(1) Cellular Proliferation. *A careful analysis of the regulatory gene targets of Kaiso/p120ctn revealed the repression of c-Myc and cyclin D1 [28, 29], which are known to be involved in cell proliferation and metastasis [15]. Data from our laboratory have consistently confirmed suspicions of the active participation of Kaiso in controlling cell proliferation, particularly in CML. Kaiso knockdown significantly enhances cancer cell proliferation and leads to a 100-fold increase in the expression of stem cell factor (SCF), which has a well-known role in cell survival and proliferation. Additionally, Kaiso knockdown in leukemia cells induces a substantial decrease in PU-1 expression, which is considered a fundamental factor in the transition to the leukemic state because a minimal reduction in PU-1 levels is sufficient to elicit a preleukemic state and promote the development of leukemia [20]. We speculate that Kaiso, which is usually expressed in epiblast cells, inhibits cell proliferation (presence of Kaiso in the nucleus) and that its transport to the cytoplasmic compartment (inducing the expression of proliferation genes) might be crucial in cells that are destined to undergo the EMT during gastrulation (Figures 1 and 2).

*(2) Cancer Cell Invasion and Migration. *One of the most important findings of the direct involvement of Kaiso in processes such as cell invasion and migration is its ability to inhibit the expression of the matrix metalloproteinase-7 (MMP7; also known as matrilysin) gene [30, 31]. The MMP7 gene plays a significant role in tumor cell invasion and metastasis due to its ability to degrade components of the extracellular matrix (ECM) and basement membrane (BM) [32]. Therefore, we hypothesize that nuclear Kaiso can control the invasive process after hypoblast substitution during gastrulation (Figures 1 and 2), and the displacement of Kaiso to the cytoplasm suggests an induction of MMP7 expression in the cellular context. This hypothesis is reinforced by the finding that Kaiso/p120ctn, particularly p120ctn, also acts as a metastasis suppressor [27]. A review of the experimental data showed that p120ctn is frequently altered and/or lost in colon, bladder, stomach, breast, prostate, lung, and pancreatic cancer [33]. However, cadherins are degraded by a posttranslational mechanism in the absence of p120ctn [34–36], which is required for cells that will initiate a migratory process. Therefore, both the cytoplasmic stability of the p120ctn/E-cadherin complex and nuclear Kaiso are necessary conditions in various states, such as cells that have already undergone the mesenchymal-epithelial transition (MET) process (Figures 1 and 2), reinforcing our hypothesis that the MET and EMT might directly depend on a change in the intracellular localization of Kaiso in response to specific environmental stimuli. Curiously, Kaiso has also been implicated in the control of many key cellular migration events due to its role in Wnt11 repression. The treatment of a nontransformed rat small intestinal epithelial cell line (IEC6) with Wnt11 inhibits E-cadherin expression and stimulates cell migration and contact-independent cell growth [37]. Therefore, we must determine whether the translocation of Kaiso/p120ctn to the nucleus stabilizes cells that have completed the MET, induces E-cadherin expression, decreases both MMP7 expression and cell migration and ultimately promotes the formation of the endoderm.

*(3) Cellular Differentiation Control Interconnected with the Adherens Junctions*. In accordance with the theoretical framework proposed by our hypothesis (Figure 1), Kaiso and p120ctn might also be indisputably engaged in maintenance of the differentiated state. This notion has also been confirmed experimentally because the knockdown of Kaiso/p120ctn significantly downregulates the expression of C/EBP*α* (a master regulator of stem cell homeostasis and cell differentiation), increases the expression of C/MyB (a differentiation blocker) and decreases the expression of Wnt11 (cellular differentiation factor) [20]. Another explanation for these results is a direct interaction of Kaiso/p120ctn with the adherens junction and the participation of the resulting Kaiso/p120ctm-adherens junction complex as a docking platform for many transcription factors that control both cellular proliferation and differentiation. As described in a subsequent section, the inhibition of Kaiso/p120ctn function affects cadherin stability and directly affects the function of prodifferentiation and proproliferation genes, such as *β*-catenin and YAP1.

## 3. Kaiso in the Embryological Model of Cancer

Once described that Kaiso pathway meets the minimum conditions for an EDP, in this section and the respective subsections, we are going to explain how this EDP is configured as an explanatory formula for each key aspect of the embryological cancer model [1], and they are going to be addressed in the following order: (1) Kaiso establishes an epigenetic control mechanism with proteins from the polycomb group that explains both gastrulation onset and cancer disease onset. (2) The simultaneous regulation of different processes exerted by Kaiso's protein during embryogenesis could explain the differentiation of the three embryonic germ layers during gastrulation. (3) Kaiso interacts directly with the adherens junctions and in this way explains gastrulation by reinforcing the relevant role of this pathway in an embryological model of cancer. (4) Finally, the specific regulation exerted by Kaiso on MMP7 explains the differences in invasiveness and mortality of endodermal origin cancers.

### 3.1. Role of Kaiso in the Embryological Model of Cancer: Establishing an Epigenetic Control Mechanism That Regulates the Expression of the Polycomb Group Protein (PcG) Suz12

One of the assumptions that support our hypothesis is the establishment of an epigenetic control mechanism, and as shown below, previous studies have found direct regulatory effects for Wnt11 and Wnt5a on Suz12 [38] and for Suz12 on Kaiso [39]. This feedback loop might have important consequences for the dynamics and establishment of gastrulation and the initiation of cancer.

**Figure 3:**
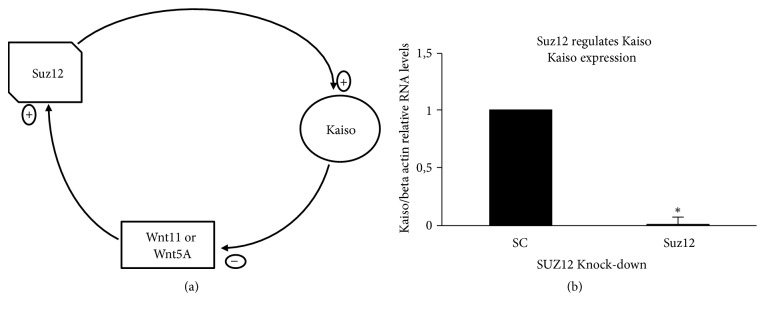
Regulatory loop among Kaiso, Wnt11/5a and Suz12. (a) The genes Wnt11 and Wnt5a upregulate Suz12 expression (suppressor of zeste 12) acting in the nucleus as transcriptional factors. Kaiso and TCF/LEF cooperate to repress the transcription of Wnt11 and Suz12 activate the transcription of Kaiso. This regulatory loop proposed could be reinforced by other nonclassical-Pc-functions as, for example: (i) EZH2 physically interacts directly with *β*-catenin, functionally improves the Wnt target gene expression and phenotypically leads to the overexpression of c-Myc and cyclin D1 [54, 55], (ii) BMI1 might also act as an activator of WNT pathway by repressing Dickkopf (DKK), and its negative regulation results in upregulation of c-Myc that participate in a positive feedback loop, activating the transcription of BMI1 [56], and (iii) the overexpression of WNT/*β*-catenin signaling can also lead to increase Wnt11 expression [58–60]. (b) Expression analysis of Kaiso in Suz12 knockdown cells. K562 cells were transfected with siRNA-Suz12 (25nM). Twenty-four hours later, RNA was isolated and subjected to Real Time RT-PCR to quantify expression of Kaiso after normalization to *β*-actin and compared to the scrambled knockdown cells. Data were expressed as mean ± standard deviation (SD). Columns, mean (n = 3); error bars, SD; *∗*p < 0.001.

A study conducted by Pizzatti et al. (2010) found that both Wnt5a and Wnt11 upregulate Suz12 expression by acting as transcription factors in the cell nucleus [38] (Figure 3(a); [40–45]). However, Wnt11 is one of several *β*-catenin/TCF-target genes that also contains a putative Kaiso-binding site in its promoter region, which suggests that Kaiso and TCF/LEF cooperate to repress Wnt11 transcription [28] (Figure 3(a)). Additionally, the knockdown of Suz12 with a small interfering RNA (siRNA) significantly decreases the expression of Kaiso in K562 cells by 98%, suggesting that at least in the context of a human erythroleukemic cell line, Suz12 exerts a positive regulatory effect on Kaiso [39] (Figure 3(b)). This regulatory loop might be extremely crucial in the control of cellular homeostasis because the levels of Suz12, Wnt11, and Kaiso need to be properly maintained during embryogenesis [39]. An apparently inconsistent finding with the well-established silencing function of PRC complexes is that the knockdown of EZH2 with a siRNA produces a significant decrease, rather than an increase, in G1/S-expressed cyclins [46], which suggests that the PRC2 complex might induce gene expression through an uncharacterized independent domain [47, 48].

As mentioned above, a critical event in the proposed theoretical framework is the intracellular relocation of Kaiso. An internal or external change in the embryo could shift Kaiso into the cytoplasmic compartment, which might trigger a cascade of events that ultimately lead to a sudden increase in the expression of Wnt11 and Suz12, potentiating their activities, specifically in cells that migrate from the primitive streak at the onset of gastrulation. One of the attractive features of this model is that it reconciles some published data on noncanonical Wnt pathways and PcG proteins and their connections (or interconnections) with the differentiation program in embryonic development (ED). Subsequently, during gastrulation, PcG proteins and Wnt11 participate in regulating the differentiation program [49–51] in primordial pluripotent embryonic stem cells, which show temporal activation or repression of specific genes, and the resulting temporal pattern defines the identify and function of these cells [52]. Furthermore, PcG proteins enable these differentiated adult cells to maintain their characteristic gene expression patterns and thereby contribute to cellular fate and memory [53]. This process might be a critical moment (a localized crisis) that allows the guidance of SC differentiation into the new mesenchymal layer and ultimately results in the robust consolidation of gastrulation.

In contrast, during the MET, Kaiso returns to the nucleus, and the expression of both Wnt11 and Suz12 decreases to the initial equilibrium levels. These alterations might represent an essential condition at the end of gastrulation to stop this process, but cells have already acquired at least two different fates or embryonic lineages over time and maintain their cellular identity and self-renewal through subsequent cell divisions. The present model also reconciles some published data on cancer, which indicate that, in several human cancer cells, Kaiso is found in the cytoplasm and Suz12 or EZH2 is overexpressed in the nucleus [19, 38, 54–57].

### 3.2. Role of Kaiso in the Embryological Model of Cancer: Explaining Gastrulation

According to our proposed hypothesis (previously described in [1]), Kaiso is shifted/displaced into the cytoplasmic compartment, but this occurs exclusively in the epiblast cells that will form the primitive streak, and this critical moment during embryonic development is indicated by an asterisk in Figure 2. The displacement of Kaiso from the nuclear compartment to the cytoplasm might be important for allowing the loss of cadherins, initiating basement membrane disruption, promoting cell proliferation and migration, and controlling cell differentiation. In particular, this latter process is very important for determining the fate of the migratory cells that are moving away from the primitive streak (mesoendodermal lineage) and express the same regulatory genes [58], and the final fates of these cells in the embryo are the mesodermal or endodermal lineages [59] (Figure 2). Nevertheless, the intracellular repositioning of Kaiso into the cytoplasm must be perfectly controlled in the epiblast microenvironment, possibly by Spemann organizer signals, cytoplasmic determinants or factors that are induced by the entry of sperm and that only trigger the EMT process in the primitive streak region [60–62].

Importantly, delaminated mesoendodermal cells are undergoing a differentiation program (a globally open chromatin state) in a temporal sequence, and consistent with embryonic events, these cells will be challenged to interact with different environments. For simplicity, we emphasize that, in the primitive streak region, where cells are delaminating, two different types of interactions are possible: only with the hypoblast exactly in the middle region of the embryo or simultaneously with the hypoblast and the extra-embryonic mesoderm in the lateral ends of the embryonic disc. The mesenchymal cells (mesoendodermal lineage) interpret these microenvironments in two different manners depending on their developmental history after primitive streak delamination. Thus, these two embryonic contexts will contribute to a change in the epigenetic state [63] and create different patterns of transcriptional regulation [64, 65] that will allow the efficient differentiation of mesodermal and endodermal lineages, as described above.

In Figure 2 (two asterisks), we intend to show the precise moment of separation between the two different lineages, and this moment is represented by the translocation of Kaiso to the nucleus, which results in the inhibition of proliferation, migration, and differentiation. We assume that Kaiso translocation will have a fundamental influence by helping close the chromatin and not allowing external factors to interfere with gene expression in these cells, and as a result, Kaiso translocation contributes to the process known as differentiation consolidation.

In contrast, one of the most important differences between mesodermal and endodermal cells is their invasive capacity (Figure 2). During gastrulation, the formation of tissues of the mesoderm lineage occurs via a direct interaction between neighboring cells (or between a cell and the ECM) or by the incorporation of new cells into aggregates of cells that will make their own BM and in turn initiate the formation of the future mesoderm [86, 87]. Nevertheless, the invasive capacity of cells of the endodermal lineage is guided by displacement via the disruption and creation of a new BM, which would result in the establishment of a new epithelium that will form the definitive endoderm [88] (Figure 2). This difference was clearly established during gastrulation and will have profound implications for the future appearance of cancer in humans. This hypothesis is supported by experimental results showing that different mechanisms control BM remodeling in mesodermal and endodermal cells [89].

Although mesodermal and endodermal cells are derived from epiblasts and migrate through the primitive streak, researchers have not clearly determined when or how the cells “decide” between these two different scenarios. These cells might be instructed to form the mesoderm or endoderm before or during migration through the primitive streak or remain multipotent (mesoendodermal lineage) before interacting with an embryonic microenvironment that promotes one cell fate over another [90]. The latter proposal suggests that a common signaling pathway initially specifies mesoendodermal cells and that a progressive sequence of determinative events will then culminate in the segregation of the endoderm and the mesoderm into morphologically distinct germ layers [59]. In zebrafish, forkhead-domain 2 protein (which is required for the specification of the foregut and midgut endoderm) is already expressed before endodermal precursors become morphologically distinct, and this finding is providing additional evidence for a common mesoendodermal precursor [59, 91]. Consistent with the theoretical framework proposed by our hypothesis, the fates of both endodermal and mesodermal cells might be determined before Kaiso translocates to the nucleus.

Finally, the signals involved in the cell fate-determining process during gastrulation are possibly directed by the anteroposterior axis [92], which is consistent with the observations that the primitive streak appears in the posterior region of the embryo and that gastrulation decisively contributes to the establishment of the anteroposterior axis. The reported experimental data are also consistent with this hypothesis because the first cells to invade and replace the hypoblast originate from the esophagus, lung, stomach, and liver in vertebrates [88], which provides a strong indication for an influence of the anteroposterior axis on cells that will undergo the MET and form the endoderm. Thus, we speculate that a regulatory mechanism involving Kaiso compartmentalization is responsible for the fine-tuning of the two critical periods of gastrulation (delamination and invasion) discussed extensively above. We predict that the embryonic microenvironment exerts a strong influence on the translocation of Kaiso from the nucleus to the cytoplasm and vice versa. Because these processes also involve the generation and dismantling of adherens junctions, we will further discuss the interconnection with Kaiso in the conceptual framework of our theoretical model.

### 3.3. Role of Kaiso in the Embryological Model of Cancer: Adapting the Embryological Model of Cancer to the New Reality of the Adherens Junctions

The following sections provide a brief description of significant developments regarding adherens junctions, which as molecular docking platforms and can gather an extensive set of transcription factors that regulate processes such as cell differentiation, proliferation, migration, and invasion. Therefore, an embryological model of cancer that is not directly mediated by cadherins is impossible to imagine. Additionally, because cadherins play an integral role in noncanonical Wnt pathways, these proteins fundamentally participate in the simultaneous regulation of the processes required for gastrulation.

The vast majority of metazoan cells spend most of their lives in close proximity to neighboring cells, and these cells must use a variety of communication tools to organize tissue activities and establish collaboration patterns between associated cells. One key to the cohesive and stable association of cells is to prevent their proliferation and thus avoid their accumulation or disorganization. A well-established model of this control mechanism is called “contact inhibition”, in which intercellular adhesion mechanisms directly inhibit cell motility and proliferation [93–95]. Cancer cells generally do not follow this biological rule. However, the experimental results reported by Pierce show that cell-cell contact might be responsible for inhibiting the tumor behavior of embryonal carcinoma cells transferred into mouse blastocysts [96–98]. These findings are compatible with the control of metastasis by contact inhibition, which potentially involves cadherins and integrins on the cell surface of embryonic cells.

Cadherins are critical regulators of ED [99, 100] and the homeostasis of adult tissues and cancer cells [101, 102]. E-cadherin mediates cell-to-cell adhesion through Ca2+-dependent homophilic interactions, and its cytoplasmic domains are connected with several catenins. The latter proteins are responsible for mediating the association between the cytoskeleton and the plasma membrane [103–105] and initiate several intracellular signaling mechanisms [66, 106]. Additionally, a loss of E-cadherin expression through genetic or epigenetic alterations promotes tumor progression and metastasis [107–110]. In contrast, the overexpression of E-cadherin in metastatic cancer cells prevents tumor progression and invasion [107, 111–115], not only through adhesive functions but also due to inhibition of cell growth signaling [115, 116] mediated by tyrosine kinase receptors (RTKs) or Src family kinases [116].

**Figure 4:**
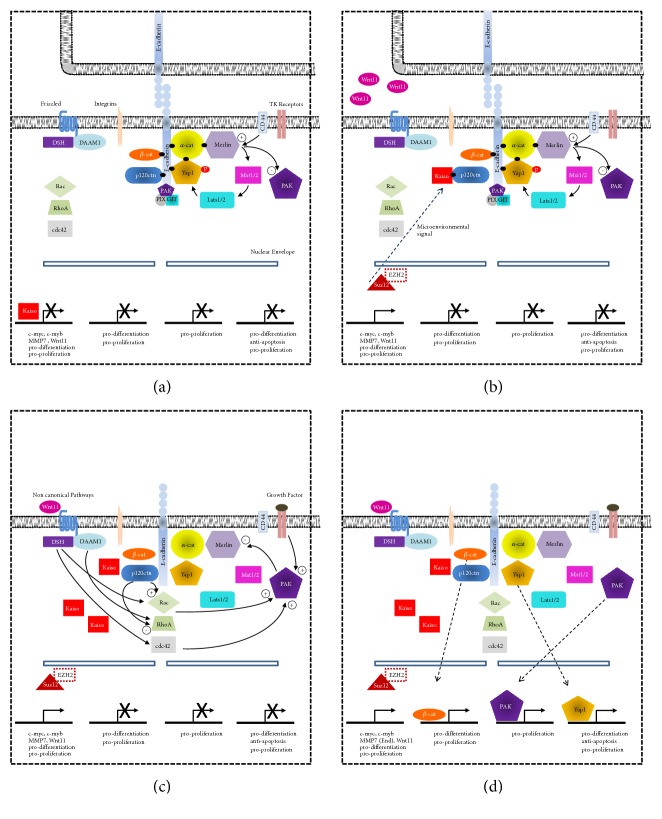
The embryonic model of cancer in the new context of adherens junctions. (a) E-cadherin as a functional platform that acts as both anchoring components of the canonical and noncanonical Wnt pathways and the Hippo signaling pathway and interacting with NF2/Merlin, Rho GTPases, and PAK-PIX-GIT. E-cadherin and *α*-catenin act as direct regulators of YAP1 and its homologue TAZ [66–68]. The canonical Hippo pathways [69], in vertebrate cells, act sequentially with MST1/2 and Lats1/2 [70, 71] to regulate the phosphorylation state of Yap1 (P, phosphate). Phosphorylation of YAP1 by Lat1/2 mediates sequestration of YAP1 in the cytoplasm while unphosphorylated YAP1 is translocated into the nucleus, where it drives the expression of genes that promote cell proliferation [71]. *α*-catenin depletion or deletion in keratinocytes shifts Yap1 into the nucleus and elevates nuclear Yap1 activity, thereby favoring cell proliferation. NF2/Merlin plays an inducer role (+) of the contact inhibition by the interaction with cadherins and catenins [72–76]. When activated, NF2/Merlin acts as a growth suppressor by inducing contact inhibition. An inducer signal is also obtained from CD44. There is evidence that Pak1 and Merlin interact reciprocally upon one other, such that Merlin also inhibits Pak1 function and suppresses the activation and the recruitment of Rac1 to the plasma membrane, which may be part of the mechanism by which Merlin regulates contact inhibition [73, 77–81]. Rho GTPases can be activated by growth factor-activated receptor or in response to cell-cell adhesion complex or cell-matrix adhesion receptors [82]. Rho GTPases participate in bidirectional signaling with both cadherins [83] and integrins [84]. In this initial context, Kaiso is found in the nucleus of the cell. (b) Kaiso in response to microenvironment signals is translocated to the cytoplasmic compartment. The immediate effect is to increase the expression of Wnt11 and Suz12 and the PCR2 complex would be involved in the repression of E-cadherin. Cytoplasmic Kaiso interacts with p120ctn and begins to destabilize the cell-cell adhesion complex. (c) The binding of Wnt to the Frizzled receptor leads to activation of Rho GTPases and PAK. p120ctn is a positive regulator of Rac1 [85] and thereby contributes to an alternative regulation of the recruitment of Rac1 to the cell-cell junctions. PAK represses NF2/Merlin and would contribute to destabilize the cell-cell adhesion complex, releasing Yap1, *β*-catenin, and the PAK-PIX-GIT complex. (d) YAP1, Pak1, and *β*-catenin, in the nucleus of the cell, activate proteins involved in processes such as cell proliferation and cell differentiation. DSH (Dishevelled); DAAM1 (Dishevelled Associated Activator of Morphogenesis 1); RAC, RhoA, and cdc42 (GTPases); p120ctn (p120 catenin); *β*-cat (*β*-catenin); *α*-cat (*α*-catenin); YAP1 (Yes-associated protein-1); PAK (p21-activated kinase); PIX (p21-activated kinase- (PAK-) interacting exchange factor); GIT (G-protein-coupled receptor-kinase-interacting protein); Lats1/2 (Large Tumor Suppressor 1 and 2, also known as Warts); Mst1/2 (Mammalian Sterile20-like 1 and 2); TK receptors (Tyrosine kinase receptors); CD44 (receptor for hyaluronic acid); Frizzled (family of G protein-coupled receptor proteins that serves as receptors in the Wnt signaling pathway).

In recent years, the “normal” view concerning the cellular roles of adherens junctions in the cell cycle and migration has been drastically altered, and our understanding of their regulatory functions has evolved to include the control of cell differentiation and the critical regulation of all processes required for induction of the EMT. Consistent with this hypothesis, the cytoplasmic domain of E-cadherin is currently considered a regulatory complex or an extremely rich cytoplasmic platform for regulating gene expression (Figure 4(a); [66–84]) that finely modulates diverse cellular functions, including cytoplasmic sequestration of Yes-associated protein 1 (YAP1) (cell-cycle control) [66], the binding of tumor suppressors, such as Kaiso/p120ctn [117–119] and NF2/Merlin (cell-cycle and differentiation control) [76], the incorporation of proteins, such as Rho GTPases (Rac, RhoA, and Cdc42) [120–122] and p21-activated kinase (Pak1) (cell-movement control) [123–126], into the adhesion complex, and the receipt and response to activated proteins, such as Rho GTPases (Rac, RhoA, and Cdc42) [82, 83] and Pak1. All these functions and the stability of the complex depend on relevant microenvironmental stimuli related to the Kaiso-P120ctn complex [23].

#### 3.3.1. A Different Facet of the Cell-Cell Adhesion Complex: Cellular Differentiation

One of the most interesting aspects of this cell-cell adhesion complex is that some of the recruited components are directly involved in controlling both the cell cycle and differentiation. These processes are closely linked to various developmental contexts (Figure 4(a)). For instance, some proteins that are anchored to the cytoplasmic tail of E-cadherin, as mentioned above, are involved in the control of cell differentiation. Thus, the Kaiso-p120ctn complex appears to regulate several genes that are directly involved in hematopoietic cell differentiation [20]. Furthermore, YAP1 overexpression in the mouse intestine or early chick neural tube promotes progenitor/stem cell expansion and the loss of differentiated cells [127, 128]. Additionally, in cell culture models used to study muscle cell or epidermal keratinocyte differentiation, YAP1 hyperactivation produces a defect in the terminal differentiation of these cells [129, 130]. Finally, the BMP signaling pathway activates YAP/TAZ to regulate mesenchymal cell differentiation [131]. Thus, these data support the hypothesis that E-cadherin functions as a platform that might regulate the transition of an adequate number of cells from the proliferative state to the quiescent state and ensure proper cell differentiation.

Notably, nuclear effectors of the Hippo signaling pathway, such as YAP and TAZ, represent attractive points of crosstalk between several different intracellular signaling pathways. For instance, Sonic hedgehog signaling serves as a positive transcriptional regulator of YAP1 in cerebellar granule neural precursors [132]. Additionally, YAP and TAZ might control the signal transduction of BMP and TGF-ß through canonical Smad-dependent pathways [133, 134], whereas a recent study by Varelas et al. showed that the Lats1/2-phosphorylated cytoplasmic TAZ protein inhibits Wnt signaling through a direct physical interaction with the Dishevelled protein [135]. These studies indicate that a complex signaling network underlies cell-cell adhesion, and this complexity reinforces an emerging picture of E-cadherin as a signaling module that integrates several types of intracellular stimuli, possibly in a context-dependent manner.

#### 3.3.2. Explaining the Occurrence of EMT in the Context of Adherens Junctions

In our model, the cytoplasmic localization of Kaiso can trigger events that reduce the amount of E-cadherin protein expressed on the cell surface. The first event refers to the role of p120ctn as an essential regulator of E-cadherin stability [34, 35]. The downregulation of p120ctn in cultured cells directly induces E-cadherin degradation [35]. Thus, p120ctn influences cell adhesion strength by controlling the availability of E-cadherin at the cell membrane (see the review by Reynolds and Roczniak-Ferguson [119]). Therefore, the cytoplasmic interaction between Kaiso and p120ctn (removing p120ctn from the adhesion complex) could trigger the first event that initiates the EMT process and thereby favor cell migration (Figure 4(b)). The second event is the well-known negative regulatory effect of some PcG proteins in the PRC2 complex on E-cadherin transcription [136]. Subsequently, the disruption of the regulatory loop between Kaiso, Wnt5a/Wnt11, and Suz12 induced by the reallocation of Kaiso increases the expression of proteins involved in the noncanonical Wnt pathway and PRC2 complex, which could help consolidate the initiation of the EMT during embryogenesis (Figures 2 and 4(b)).

The continuity of the EMT process is maintained through sequential steps that start with the Frizzled receptor and end with E-cadherin (loss of cell-cell adhesion). The initial step is the increased expression of Wnt11 and its secretion into the extracellular milieu, and this secretion, through the Frizzled (Fz) receptor, activates the small GTPase Rho and Pak1 (Figure 4(c); [85]). The next major step in the process is NF2/Merlin repression by Pak1, which might lead to destabilization of the cell-cell adhesion complex and ensure the release of YAP1 to subsequently promote the translocation of *β*-catenin and Pak1 to the nucleus and thereby induce the expression of genes involved in proliferation and cellular differentiation (Figure 4(d)). Moreover, the overexpression of PcG proteins also decisively contributes to the consolidation of mesoendodermal differentiation and drives differentiation toward a particular lineage, thereby establishing mesodermal or endodermal lineages throughout the process [53]. The role of PcG proteins in regulating gene expression, specifically in SC lineage choice, commitment and differentiation, might be divided into classical and nonclassical functions. The classical function includes the trimethylation of histone H3 lysine 27 (H3K27me3) by PRC2 [137, 138]. In contrast, the nonclassical function involves the activation of Wnt/*β*-catenin signaling by EZH2 [40, 41] or BMI1 [42]. The latter two nonclassical functions are compatible with a hypothetical model explaining the endodermal differentiation activated by *β*-catenin [139], which is also consistent with our embryological model (Figure 4(b)). The histone H3K27me3 demethylases KDM6A and KDM6B might be responsible for definitive endoderm differentiation during the late gastrulation stage [139].

Finally, in our model, we must also consider numerous growth factors that are known to influence mesodermal and endodermal differentiation, including fibroblast growth factor (FGF), transforming growth factor beta (TGF*β*), Wnt growth factor, and retinoic acid (Figures 4(c) and 4(d)) (see the review by Wells and Melton [90]). For instance, bone morphogenetic protein 4 (BMP4) is essential for mesoderm differentiation during mouse gastrulation [140], and in vitro studies using human embryonic stem cell lines have revealed relevant roles for Wnt, Activin A, Nodal, BMPs (see the review by Lewis and Tam [141]), and small molecules [142] in endodermal differentiation.

### 3.4. Role of Kaiso in the Embryological Model of Cancer: The Transcriptional Control of Matrilysin in the Endoderm Layer

Matrix metalloproteinases (MMPs) are a family of zinc-dependent proteolytic enzymes that degrade most of the components of the ECM. The MMP-mediated degradation of ECM occurs during many normal physiological processes, such as tissue morphogenesis, angiogenesis, bone remodeling, differentiation, and wound healing [143]. MMP family members have also been implicated in cancer, specifically by contributing to tumor progression, cancer invasion, and metastasis [144]. Of particular relevance is MMP-7 (EC 3.4.24.23), which is expressed at high levels almost exclusively by glandular epithelial cells of diverse embryonic origins, such as the human cycling endometrium, Paneth cells of small intestinal crypts, normal mature skin, the glandular epithelium of the mammary gland, parotid glands, the pancreas, the liver, the prostate, and the lung [145, 146]. Additionally, MMP7 degrades many ECM proteins, including proteoglycans, fibronectin, entactin, laminin, gelatin, and elastin [143].

One of the most surprising findings is that matrilysin is almost exclusively expressed in epithelial tumors of endodermal origin (with breast tissue being the only exception) [146], whereas the majority of other MMPs are predominantly expressed in normal stromal cells immediately adjacent to tumor tissue [147]. Indeed, the transcriptional repression of matrilysin mediated by Kaiso was particularly observed in using gastric epithelial cells in vitro and in vivo [31]. In this context, endodermal cells might retain Kaiso-dependent transcriptional repression of MMP7 during the EMT process, leading to hypoblast invasion and the formation of the new definitive endoderm. The hypoblast does not have a true basal membrane, making it a more sensitive tissue to invasion (during embryogenesis). Thus, the SCs of endodermal origin that are in the process of invading the hypoblast will likely use MMP7 to promote the invasion process via destruction of E-cadherin at the cell surface, or once the definitive endoderm is established, MMP7 might mediate the necessary remodeling of the new BM. Based on this reasoning, more recent studies corroborate the hypothesis that E-cadherin might be a new substrate for matrilysin, which suggests that cleavage of the E-cadherin ectodomain might be required for lung epithelial repair [148] or as a new mechanism to explain the invasive potential of oral squamous cell carcinomas [149] and prostate cancer [150].

Therefore, in our embryological model of cancer, pluripotent epiblast SCs provide the link between Kaiso and MMP7, which might help some epiblast cells trigger the EMT process, and this relationship might be retained in mesoendodermal cells “in transit” during gastrulation. Soon afterward, the consolidation of specific mesoderm and ectoderm cell populations leads to the obvious consequence of widespread genome reorganization, which suggests that Kaiso cannot obtain greater access to the transcriptional control of MMP7 gene expression in these embryonic populations. Consistent with this hypothesis, the expression of matrilysin has been observed in morphologically normal epithelial cells and fibroblasts surrounding the tumor cells in 50% of the analyzed cases of human mammary tumors [151]. Additionally, mammary epithelial tumors of ectodermal origin appear to need significant support from stromal cells to initiate the invasion process [152]. Thus, the transcriptional repression of MMP7 is independent of Kaiso in mesoderm and ectoderm cells but might have similar effects on adherens junctions [153, 154]. Finally, the highly invasive potential of endodermal cells might be a consequence of the direct relationship between the Kaiso-dependent transcriptional control of MMP7 and the cell-cell adhesion complex.

## 4. Concluding Remarks

### 4.1. The Weakness of Our Experimental Models: Controversy Concerning the Role of Kaiso

Considering the words of Richmond Prehn, “it may be more correct to say that cancers beget mutations than it is to say that mutations beget cancers” [155], we intend to finish the article with a critical reflection and self-criticism of some of the experimental models used in studies of human cancer. First, we will provide a brief description of the cell culture models used to characterize the divergent functions of Kaiso as a tumor suppressor or oncogene [20, 156, 157] that negatively impact the advances in cancer research as a whole. The cell culture models used include the K562 cell line [158], which was obtained from a patient with CML in the blast crisis phase, the DU-145 [159] and PC-3 cell lines [160], which were obtained from patients with prostate cancer, and the MCF-7 (nonmetastatic) [161], MDA-MB-468 (few metastases) [162], and MDA-MB-231 (highly metastatic) [162] cell lines, which are breast cancer cell lines.

Analyses of the composition and number of chromosomes in prostate carcinoma cell lines have revealed that DU145 cells have a hypotriploid karyotype with 62 (range, 57–65) chromosomes [163], and the two different PC-3 subclones identified (hypotriploid and hypopentaploid) have 58 (range, 57–61) and 113 (range, 112–114) chromosomes, respectively [164]. DU145 and PC-3 cells exhibit some structural alterations involving chromosomes 1, 2, 4, 6, 10, 15, and 16 and translocations of chromosomes 1, 4, 6, 15, and 16 [163]. In contrast, analyses of mammary cell lines have revealed that MCF-7 cells have 88 chromosomes [161], MDA-MB-231 cells have 64 chromosomes [162], and MBD-MB-468 cells have a modal number of 60 chromosomes, with 42 types of aberrations (11 numerical and 31 structural) [165]. Finally, unsurprisingly, chromosomal analyses of K562 cells have revealed a hypotriploid karyotype with 66-72 chromosomes that contain various numerical and structural aberrations [166].

Surprisingly, the original patient sample used to establish the K562 cell line had 46 chromosomes. However, after ten passages, most of the cells had a modal number of 50-52 chromosomes. By passage 110, two subclones were derived, and the number of chromosomes had increased. One line had a near-triploid karyotype with 69-73 chromosomes, whereas the second line had a near-tetraploid karyotype with 90-96 chromosomes [166]. Furthermore, six months [161] and 90 passages in vitro over a period of 2 years [159] were needed to obtain DU-145 cells from a primary culture of MCF-7 cells.

Thus, the cells used to study the function of Kaiso show marked differences in their genetic repertoire and stages of oncogenic transformation. Appropriate comparisons between these cancer cells are impossible without understanding the real impact of different chromosomal aberrations on the homeostasis of each of these cell lines. What are the consequences of these chromosomal aberrations? We can only obtain the answer to that question if the entire scientific community performs in-depth studies aiming to elucidate the biology of each of these cell lines. Would these investigations be worth the effort? What are we studying? Are we studying the influence of cancer on chromosomal instability? An advocate of the somatic mutation theory of cancer could fully justify exploring the meaning of these aberrations to understand how to apply the obtained knowledge to the development of a new specific therapy for cancer.

We provide some illustrative examples to refute the latter justification. First, human embryonic stem cells (hESCs), which are expected to display a stable euploid karyotype, have, in the last decade, exhibited several chromosomal aberrations that were systematically described in all these cell lines during their maintenance in vitro [167–171]. The in vitro culture conditions or long-term culture have frequently been proposed as possible factors implicated in the acquisition of chromosomal abnormalities. Moreover, stem cell therapy and regenerative medicine based on triggering hESC differentiation has become one of the most promising avenues of research in the areas of health and medicine since these cells were successfully established [172]. Therefore, are cancer cells in culture considered an appropriate model for cancer?

A second example shows an even more disheartening perspective because the majority of cancers obtained from patient biopsies are in fact genetically unstable at either the nucleotide or the chromosome level [173, 174] and are also epigenetically altered [175, 176]. A further complication that results from the dynamic nature of tumors is the development of cancer occurs over decades [177], and the chronological history of these instabilities and their contribution to tumor development cannot be determined. In contrast, embryonic models are rarely explored and less well known. For instance, cancer cells show phenotype reversibility when placed within the early embryo, even under conditions favoring chromosomal instability. Thus, although most of the teratocarcinoma cell lines cultured “in vitro” do not in fact have a normal number of chromosomes [178–180], the introduction of these cells into blastocyst-stage embryos allows clonal propagation of the carcinoma cells and the production of teratoma-free animals with many tumor-derived normal tissues [181, 182]. Following the same line of thought, cell-cell contact with the trophectoderm or inner cell mass (ICM) might be responsible for inhibiting the metastatic behavior of two different teratocarcinoma cell lines (402AX and EC247) transplanted in mouse blastocysts [96–98]. Moreover, the neoplastic phenotype of hepatic carcinoma cells is reversed within one month after the cells are transplanted into young rat livers, but these cells grew progressively after transplantation into older rats [183]; these effects are similar to those obtained for embryonic skin on the growth of melanoma tumors [184].

### 4.2. Embryological Foundations of Cancer

A crucial factor limiting the consolidation of an embryological model of cancer is the difficulty in establishing an embryological model that would enable research on cell movements and developmental pathways in humans. The limitations include bioethical issues [185–188] and difficulties associated with embryo handling and observation after implantation in the mother's womb. The mouse model was initially a promising model because of its resemblance to humans. However, several lines of evidence obtained in recent years have shown significant differences between humans and mice that eliminate the possibility of establishing parameters for reliable comparisons at the cell and molecular levels [189–192].

Despite these differences and the lack of an embryonic model of study, embryologists have formulated explanatory cancer models in the twentieth century within a conceptual framework that directly links the phenomenon of cancer to cell differentiation and a hierarchy level focused on the cell that restricts the cancer aspect specifically to stem cells in an adult individual [180, 193–195]. However, the embryological tradition has historically adopted an understanding of cancer at a different hierarchical level and attributed the appearance of cancer to the loss of control forces at the tissue level [196]. Surprisingly, “the controlling forces from which the cancerous growth has escaped” [196] show incredible resonance with the current strengthening hypothesis proposed in the twenty-first century of the role of cadherins in cancer and their close relationship with transcription factors that control differentiation and cell proliferation. However, cell migration and invasion are also properties that are controlled by adherens junctions, and in particularly, cell invasiveness is controlled through direct regulation by noncanonical Wnt pathways, as described extensively in this article.

Therefore, in the twenty-first century, two significant features are consolidated within an embryological conceptual framework of cancer: a vision of the cancer phenomenon included within the cell differentiation process [193, 194] and a change in the cancer hierarchical level to a tissue level, as was proposed by several researchers [1, 197, 198]. These two aspects are fundamental to our theoretical proposal of the relationship established between Kaiso and matrilysin in the embryonic endoderm layer and potentially explain why cancers of endodermal origin are more invasive and deadlier in the human population. Finally, using the embryological cancer hypothesis, we hope to help redirect and generate changes in cancer research.

## References

[1] Cofre J., Abdelhay E. (2017). Cancer is to embryology as mutation is to genetics: hypothesis of the cancer as embryological phenomenon. *The Scientific World Journal*.

[2] Waddington C. H. (1968). Towards a theoretical biology. *Nature*.

[3] Jablonka E., Lamb M. J. (2002). The changing concept of epigenetics. *Annals of the New York Academy of Sciences*.

[4] Nanney D. L. (1958). Epigenetic control systems. *Proceedings of the National Acadamy of Sciences of the United States of America*.

[5] Clouaire T., Stancheva I. (2008). Methyl-CpG binding proteins: Specialized transcriptional repressors or structural components of chromatin?. *Cellular and Molecular Life Sciences*.

[6] Bogdanović O., Veenstra G. J. C. (2009). DNA methylation and methyl-CpG binding proteins: developmental requirements and function. *Chromosoma*.

[7] Parry L., Clarke A. R. (2011). The roles of the methyl-CpG binding proteins in cancer. *Genes & Cancer*.

[8] Sansom O. J., Maddison K., Clarke A. R. (2007). Mechanisms of disease: methyl-binding domain proteins as potential therapeutic targets in cancer. *Nature Clinical Practice Oncology*.

[9] Daniel J. M., Spring C. M., Crawford H. C., Reynolds A. B., Baig A. (2002). The p120ctn-binding partner Kaiso is a bi-modal DNA-binding protein that recognizes both a sequence-specific consensus and methylated CpG dinucleotides. *Nucleic Acids Research*.

[10] Prokhortchouk A., Hendrich B., Jørgensen H. (2001). The p120 catenin partner Kaiso is a DNA methylation-dependent transcriptional repressor. *Genes & Development*.

[11] Sasai N., Nakao M., Defossez P.-A. (2010). Sequence-specific recognition of methylated DNA by human zinc-finger proteins. *Nucleic Acids Research*.

[12] Fournier A., Sasai N., Nakao M., Defossez P.-A. (2012). The role of methyl-binding proteins in chromatin organization and epigenome maintenance. *Briefings in Functional Genomics*.

[13] Lopes E. C., Valls E., Figueroa M. E. (2008). Kaiso contributes to DNA methylation-dependent silencing of tumor suppressor genes in colon cancer cell lines. *Cancer Research*.

[14] Yoon H.-G., Chan D. W., Reynolds A. B., Qin J., Wong J. (2003). N-CoR mediates DNA methylation-dependent repression through a methyl CpG binding protein Kaiso. *Molecular Cell*.

[15] Daniel J. M. (2007). Dancing in and out of the nucleus: p120ctn and the transcription factor Kaiso. *Biochimica et Biophysica Acta (BBA) - Molecular Cell Research*.

[16] Ruzov A., Dunican D. S., Prokhortchouk A. (2004). Kaiso is a genome-wide repressor of transcription that is essential for amphibian development. *Development*.

[17] Wallingford J. B., Fraser S. E., Harland R. M. (2002). Convergent extension: the molecular control of polarized cell movement during embryonic development. *Developmental Cell*.

[18] Veeman M. T., Axelrod J. D., Moon R. T. (2003). A second canon: functions and mechanisms of *β*-catenin-independent Wnt signaling. *Developmental Cell*.

[19] Kim S. W., Park J.-I., Spring C. M. (2004). Non-canonical Wnt signals are modulated by the Kaiso transcriptional repressor and p120-catenin. *Nature Cell Biology*.

[20] Cofre J., Menezes J. R. L., Pizzatti L., Abdelhay E. (2012). Knock-down of Kaiso induces proliferation and blocks granulocytic differentiation in blast crisis of chronic myeloid leukemia. *Cancer Cell International*.

[21] Daniel J. M., Reynolds A. B. (1999). The catenin p120(ctn) interacts with Kaiso, a novel BTB/POZ domain zinc finger transcription factor. *Molecular and Cellular Biology*.

[22] Daniel J. M., Ireton R. C., Reynolds A. B. (2001). Monoclonal antibodies to Kaiso: a novel transcription factor and p120ctn-binding protein. *Hybridoma*.

[23] Soubry A., Van Hengel J., Parthoens E. (2005). Expression and nuclear location of the transcriptional repressor Kaiso is regulated by the tumor microenvironment. *Cancer Research*.

[24] Dai S.-D., Wang Y., Miao Y. (2009). Cytoplasmic Kaiso is associated with poor prognosis in non-small cell lung cancer. *BMC Cancer*.

[25] Collins T., Stone J. R., Williams A. J. (2001). All in the family: the BTB/POZ, KRAB, and SCAN domains. *Molecular and Cellular Biology*.

[26] Iioka H., Doerner S. K., Tamai K. (2009). Kaiso is a bimodal modulator for Wnt/*β*-catenin signaling. *FEBS Letters*.

[27] van Roy F. M., McCrea P. D. (2005). A role for kaiso-p120ctn complexes in cancer?. *Nature Reviews Cancer*.

[28] Park J.-I., Kim S. W., Lyons J. P. (2005). Kaiso/p120-Catenin and TCF/*β*-Catenin complexes coordinately regulate canonical Wnt gene targets. *Developmental Cell*.

[29] Donaldson N. S., Pierre C. C., Anstey M. I. (2012). Kaiso represses the cell cycle gene cyclin D1 via sequence-specific and methyl-CpG-dependent mechanisms. *PLoS ONE*.

[30] Spring C. M., Kelly K. F., O'Kelly I., Graham M., Crawford H. C., Daniel J. M. (2005). The catenin p120ctn inhibits Kaiso-mediated transcriptional repression of the *β*-catenin/TCF target gene matrilysin. *Experimental Cell Research*.

[31] Ogden S. R., Wroblewski L. E., Weydig C. (2008). p120 and Kaiso regulate Helicobacter pylori-induced expression of matrix metalloproteinase-7. *Molecular Biology of the Cell (MBoC)*.

[32] Basu S., Thorat R., Dalal S. N. (2015). MMP7 is required to mediate cell invasion and tumor formation upon plakophilin3 loss. *PLoS ONE*.

[33] Thoreson M. A., Reynolds A. B. (2002). Altered expression of the catenin p120 in human cancer: Implications for tumor progression. *Differentiation*.

[34] Ireton R. C., Davis M. A., van Hengel J. (2002). A novel role for p120 catenin in E-cadherin function. *The Journal of Cell Biology*.

[35] Davis M. A., Ireton R. C., Reynolds A. B. (2003). A core function for p120-catenin in cadherin turnover. *The Journal of Cell Biology*.

[36] Xiao K., Allison D. F., Buckley K. M. (2003). Cellular levels of p120 catenin function as a set point for cadherin expression levels in microvascular endothelial cells. *The Journal of Cell Biology*.

[37] Ouko L., Ziegler T. R., Gu L. H., Eisenberg L. M., Yang V. W. (2004). Wnt11 signaling promotes proliferation, transformation, and migration of IEC6 intestinal epithelial cells. *The Journal of Biological Chemistry*.

[38] Pizzatti L., Binato R., Cofre J. (2010). SUZ12 is a candidate target of the non-canonical WNT pathway in the progression of chronic myeloid leukemia. *Genes, Chromosomes and Cancer*.

[39] Cofre J., Abdelhay E. (2017). A feedback loop that regulates the expression of polycomb group protein Suz12 via non-canonical WNT signaling pathway in blast crisis of chronic myeloid leukemia. *Hematology and Leukemia*.

[40] Shi B., Liang J., Yang X. (2007). Integration of estrogen and Wnt signaling circuits by the polycomb group protein EZH2 in breast cancer cells. *Molecular and Cellular Biology*.

[41] Cheng A. S. L., Lau S. S., Chen Y. (2011). EZH2-mediated concordant repression of Wnt antagonists promotes *β*-catenin-dependent hepatocarcinogenesis. *Cancer Research*.

[42] Cho J.-H., Dimri M., Dimri G. P. (2013). A positive feedback loop regulates the expression of polycomb group protein BMI1 via WNT signaling pathway. *The Journal of Biological Chemistry*.

[43] Lin Z., Reierstad S., Huang C.-C., Bulun S. E. (2007). Novel estrogen receptor-*α* binding sites and estradiol target genes identified by chromatin immunoprecipitation cloning in breast cancer. *Cancer Research*.

[44] Gros J., Serralbo O., Marcelle C. (2009). WNT11 acts as a directional cue to organize the elongation of early muscle fibres. *Nature*.

[45] Dwyer M. A., Joseph J. D., Wade H. E. (2010). WNT11 expression is induced by estrogen-related receptor *α* and *β*-catenin and acts in an autocrine manner to increase cancer cell migration. *Cancer Research*.

[46] Bracken A. P., Pasini D., Capra M., Prosperini E., Colli E., Helin K. (2003). EZH2 is downstream of the pRB‐E2F pathway, essential for proliferation and amplified in cancer. *EMBO Journal*.

[47] Shearn A. (1989). The ash-1, ash-2 and trithorax genes of *Drosophila melanogaster* are functionally related. *Genetics*.

[48] LaJeunesse D., Shearn A. (1995). Trans-regulation of thoracic homeotic selector genes of the Antennapedia and bithorax complexes by the trithorax group genes: absent, small, and homeotic discs 1 and 2. *Mechanisms of Development*.

[49] Prezioso C., Orlando V. (2011). Polycomb proteins in mammalian cell differentiation and plasticity. *FEBS Letters*.

[50] Pandur P., Läsche M., Eisenberg L. M., Kühl M. (2002). Wnt-11 activation of a non-canonical Wnt signalling pathway is required for cardiogenesis. *Nature*.

[51] Terami H., Hidaka K., Katsumata T., Iio A., Morisaki T. (2004). Wnt11 facilitates embryonic stem cell differentiation to Nkx2.5-positive cardiomyocytes. *Biochemical and Biophysical Research Communications*.

[52] Sparmann A., van Lohuizen M. (2006). Polycomb silencers control cell fate, development and cancer. *Nature Reviews Cancer*.

[53] Signolet J., Hendrich B. (2015). The function of chromatin modifiers in lineage commitment and cell fate specification. *FEBS Journal*.

[54] Kleer C. G., Cao Q., Varambally S. (2003). EZH2 is a marker of aggressive breast cancer and promotes neoplastic transformation of breast epithelial cells. *Proceedings of the National Acadamy of Sciences of the United States of America*.

[55] Glinsky G. V., Berezovska O., Glinskii A. B. (2005). Microarray analysis identifies a death-from-cancer signature predicting therapy failure in patients with multiple types of cancer. *The Journal of Clinical Investigation*.

[56] Guo W.-J., Zeng M.-S., Yadav A. (2007). Mel-18 acts as a tumor suppressor by repressing Bmi-1 expression and down-regulating Akt activity in breast cancer cells. *Cancer Research*.

[57] Varambally S., Dhanasekaran S. M., Zhou M. (2002). The polycomb group protein EZH2 is involved in progression of prostate cancer. *Nature*.

[58] Hammerschmidt M., Nusslein-Volhard C. (1993). The expression of a zebrafish gene homologous to Drosophila snail suggests a conserved function in invertebrate and vertebrate gastrulation. *Development*.

[59] Warga R. M., Nüsslein-Volhard C. (1999). Origin and development of the zebrafish endoderm. *Development*.

[60] Gerhart J., Danilchik M., Doniach T., Roberts S., Rowning B., Stewart R. (1989). Cortical rotation of the Xenopus egg: Consequences for the anteroposterior pattern of embryonic dorsal development. *Development*.

[61] Miller J. R., Rowning B. A., Larabell C. A., Yang-Snyder J. A., Bates R. L., Moon R. T. (1999). Establishment of the dorsal-ventral axis in Xenopus embryos coincides with the dorsal enrichment of dishevelled that is dependent on cortical rotation. *The Journal of Cell Biology*.

[62] Piotrowska K., Zernicka-Goetz M. (2001). Role for sperm in spatial patterning of the early mouse embryo. *Nature*.

[63] Arney K. L., Fisher A. G. (2004). Epigenetic aspects of differentiation. *Journal of Cell Science*.

[64] Henry G. L., Melton D. A. (1998). Mixer, a homeobox gene required for endoderm development. *Science*.

[65] Lemaire P., Darras S., Caillol D., Kodjabachian L. (1998). A role for the vegetally expressed Xenopus gene Mix. 1 in endoderm formation and in the restriction of mesoderm to the marginal zone. *Development*.

[66] Kim N.-G., Koh E., Chen X., Gumbiner B. M. (2011). E-cadherin mediates contact inhibition of proliferation through Hippo signaling-pathway components. *Proceedings of the National Acadamy of Sciences of the United States of America*.

[67] Schlegelmilch K., Mohseni M., Kirak O. (2011). Yap1 acts downstream of *α*-catenin to control epidermal proliferation. *Cell*.

[68] Silvis M. R., Kreger B. T., Lien W. (2011). *α*-catenin is a tumor suppressor that controls cell accumulation by regulating the localization and activity of the transcriptional coactivator Yap1. *Science Signaling*.

[69] Pan D. (2010). The hippo signaling pathway in development and cancer. *Developmental Cell*.

[70] Chan E. H. Y., Nousiainen M., Chalamalasetty R. B., Schäfer A., Nigg E. A., Sillje H. H. W. (2005). The Ste20-like kinase Mst2 activates the human large tumor suppressor kinase Lats1. *Oncogene*.

[71] Zhao B., Wei X., Li W. (2007). Inactivation of YAP oncoprotein by the hippo pathway is involved in cell contact inhibition and tissue growth control. *Genes & Development*.

[72] Lallemand D., Curto M., Saotome I., Giovannini M., McClatchey A. I. (2003). NF2 deficiency promotes tumorigenesis and metastasis by destabilizing adherens junctions. *Genes & Development*.

[73] Okada T., You L., Giancotti F. G. (2007). Shedding light on Merlin's wizardry. *Trends in Cell Biology*.

[74] Curto M., Cole B. K., Lallemand D., Liu C., McClatchey A. I. (2007). Contact-dependent inhibition of EGFR signaling by Nf2/Merlin. *The Journal of Cell Biology*.

[75] McClatchey A. I., Fehon R. G. (2009). Merlin and the ERM proteins-regulators of receptor distribution and signaling at the cell cortex. *Trends in Cell Biology*.

[76] Gladden A. B., Hebert A. M., Schneeberger E. E., McClatchey A. I. (2010). The NF2 tumor suppressor, merlin, regulates epidermal development through the establishment of a junctional polarity complex. *Developmental Cell*.

[77] Kissil J. L., Johnson K. C., Eckman M. S., Jacks T. (2002). Merlin phosphorylation by p21-activated kinase 2 and effects of phosphorylation on merlin localization. *The Journal of Biological Chemistry*.

[78] Xiao G.-H., Beeser A., Chernoff J., Testa J. R. (2002). p21-activated kinase links Rac/Cdc42 signaling to merlin. *The Journal of Biological Chemistry*.

[79] Kissil J. L., Wilker E. W., Johnson K. C., Eckman M. S., Yaffe M. B., Jacks T. (2003). Merlin, the product of the Nf2 tumor suppressor gene, is an inhibitor of the p21-activated kinase, Pak1. *Molecular Cell*.

[80] Okada T., Lopez-Lago M., Giancotti F. G. (2005). Merlin/NF-2 mediates contact inhibition of growth by suppressing recruitment of Rac to the plasma membrane. *The Journal of Cell Biology*.

[81] Shaw R. J., Paez J. G., Curto M. (2001). The Nf2 tumor suppressor merlin, functions in Rac -dependent signaling. *Developmental Cell*.

[82] Burute M., Thery M. (2012). Spatial segregation between cell-cell and cell-matrix adhesions. *Current Opinion in Cell Biology*.

[83] Kaibuchi K., Kuroda S., Fukata M., Nakagawa M. (1999). Regulation of cadherin-mediated cell-cell adhesion by the rho family GTPases. *Current Opinion in Cell Biology*.

[84] Keely P., Parise L., Juliano R. (1998). Integrins and GTPases in tumour cell growth, motility and invasion. *Trends in Cell Biology*.

[85] Noren N. K., Liu B. P., Burridge K., Kreft B. (2000). p120 catenin regulates the actin cytoskeleton via RHO family GTPases. *The Journal of Cell Biology*.

[86] Tam P. P. L., Meier S., Jacobson A. G. (1982). Differentiation of the metameric pattern in the embryonic axis of the mouse: II. Somitomeric organization of the presomitic mesoderm. *Differentiation*.

[87] Tam P. P. L., Beddington R. S. P. (1987). The formation of mesodermal tissues in the mouse embryo during gastrulation and early organogenesis. *Development*.

[88] Lawson K. A., Meneses J. J., Pedersen R. A. (1986). Cell fate and cell lineage in the endoderm of the presomite mouse embryo, studied with an intracellular tracer. *Developmental Biology*.

[89] Costello I., Biondi C. A., Taylor J. M., Bikoff E. K., Robertson E. J. (2009). Smad4-dependent pathways control basement membrane deposition and endodermal cell migration at early stages of mouse development. *BMC Developmental Biology*.

[90] Wells J. M., Melton D. A. (1999). Vertebrate endoderm development. *Annual Review of Cell and Developmental Biology*.

[91] Odenthal J., Nüsslein-Volhard C. (1998). Fork head domain genes in zebrafish. *Development Genes and Evolution*.

[92] Wells J. M., Melton D. A. (2000). Early mouse endoderm is patterned by soluble factors from adjacent germ layers. *Development*.

[93] Stoker M. G. P., Rubin H. (1967). Density dependent inhibition of cell growth in culture. *Nature*.

[94] Middleton C. A. (1972). Contact inhibition of locomotion in cultures of pigmented retina epithelium. *Experimental Cell Research*.

[95] Abercrombie M. (1979). Contact inhibition and malignancy. *Nature*.

[96] Pierce G. B., Lewis S. H., Miller G. J., Moritz E., Miller P. (1979). Tumorigenicity of embryonal carcinoma as an assay to study control of malignancy by the murine blastocyst. *Proceedings of the National Acadamy of Sciences of the United States of America*.

[97] Pierce G. B., Pantazis C. G., Caldwell J. E., Wells R. S. (1982). Specificity of the control of tumor formation by the blastocyst. *Cancer Research*.

[98] Pierce G. B., Aguilar D., Hood G., Wells R. S. (1984). Trophectoderm in control of murine embryonal carcinoma. *Cancer Research*.

[99] Edelman G. M., Gallin W. J., Delouvee A., Cunningham B. A., Thiery J. P. (1983). Early epochal maps of two different cell adhesion molecules. *Proceedings of the National Acadamy of Sciences of the United States of America*.

[100] Burdsal C. A., Damsky C. H., Pedersen R. A. (1993). The role of E-cadherin and integrins in mesoderm differentiation and migration at the mammalian primitive streak. *Development*.

[101] Thiery J. P. (2002). Epithelial-mesenchymal transitions in tumor progression. *Nature Reviews Cancer*.

[102] Gumbiner B. M. (2005). Regulation of cadherin-mediated adhesion in morphogenesis. *Nature Reviews Molecular Cell Biology*.

[103] Takeichi M. (1995). Morphogenetic roles of classic cadherins. *Current Opinion in Cell Biology*.

[104] Kemler R. (1993). From cadherins to catenins: cytoplasmic protein interactions and regulation of cell adhesion. *Trends in Genetics*.

[105] Tepass U., Truong K., Godt D., Ikura M., Peifer M. (2000). Cadherins in embryonic and neural morphogenesis. *Nature Reviews Molecular Cell Biology*.

[106] Harris T. J. C., Tepass U. (2010). Adherens junctions: from molecules to morphogenesis. *Nature Reviews Molecular Cell Biology*.

[107] Behrens J., Mareel M. M., Van Roy F. M., Birchmeier W. (1989). Dissecting tumor cell invasion: epithelial cells acquire invasive properties after the loss of uvomorulin-mediated cell-cell adhesion. *The Journal of Cell Biology*.

[108] Chen W., Obrink B. (1991). Cell-cell contacts mediated by E-cadherin (uvomorulin) restrict invasive behavior of L-cells. *The Journal of Cell Biology*.

[109] Hajra K. M., Fearon E. R. (2002). Cadherin and catenin alterations in human cancer. *Genes, Chromosomes and Cancer*.

[110] Jeanes A., Gottardi C. J., Yap A. S. (2008). Cadherins and cancer: how does cadherin dysfunction promote tumor progression?. *Oncogene*.

[111] Navarro P., Gómez M., Pizarro A., Gamallo C., Quintanilla M., Cano A. (1991). A role for the E-cadherin cell-cell adhesion molecule during tumor progression of mouse epidermal carcinogenesis. *The Journal of Cell Biology*.

[112] Vleminckx K., Vakaet L., Mareel M., Fiers W., Van Roy F. (1991). Genetic manipulation of E-cadherin expression by epithelial tumor cells reveals an invasion suppressor role. *Cell*.

[113] Kim J.-B., Islam S., Kim Y. J. (2000). N-cadherin extracellular repeat 4 mediates epithelial to mesenchymal transition and increased motility. *The Journal of Cell Biology*.

[114] Wong A. S. T., Gumbiner B. M. (2003). Adhesion-independent mechanism for suppression of tumor cell invasion by E-cadherin. *The Journal of Cell Biology*.

[115] Gottardi C. J., Wong E., Gumbiner B. M. (2001). E-cadherin suppresses cellular transformation by inhibiting *β*-catenin signaling in an adhesion-independent manner. *The Journal of Cell Biology*.

[116] Perrais M., Chen X., Perez-Moreno M., Gumbiner B. M. (2007). E-cadherin homophilic ligation inhibits cell growth and epidermal growth factor receptor signaling independently of other cell interactions. *Molecular Biology of the Cell (MBoC)*.

[117] Peifer M., Yap A. S. (2003). Traffic control: p120-catenin acts as a gatekeeper to control the fate of classical cadherins in mammalian cells. *The Journal of Cell Biology*.

[118] Reynolds A. B., Carnahan R. H. Regulation of cadherin stability and turnover by p120ctn: implications in disease and cancer.

[119] Reynolds A. B., Roczniak-Ferguson A. (2004). Emerging roles for p120-catenin in cell adhesion and cancer. *Oncogene*.

[120] Settleman J. (2000). Getting in shape with Rho. *Nature Cell Biology*.

[121] Kovacs E. M., Ali R. G., McCormack A. J., Yap A. S. (2002). E-cadherin homophilic ligation directly signals through Rac and phosphatidylinositol 3-kinase to regulate adhesive contacts. *The Journal of Biological Chemistry*.

[122] Van Aelst L., Symons M. (2002). Role of Rho family GTPases in epithelial morphogenesis. *Genes & Development*.

[123] Manser E., Leung T., Salihuddin H., Zhao Z.-S., Lim L. (1994). A brain serine/threonine protein kinase activated by Cdc42 and Rac1. *Nature*.

[124] Jaffe A. B., Hall A. (2005). Rho GTPases: biochemistry and biology. *Annual Review of Cell and Developmental Biology*.

[125] Zegers M. M. P., Forget M.-A., Chernoff J., Mostov K. E., Ter Beest M. B. A., Hansen S. H. (2003). Pak1 and PIX regulate contact inhibition during epithelial wound healing. *EMBO Journal*.

[126] Zegers M. (2008). Roles of P21-activated Kinases and associated proteins in epithelial wound healing. *International Review of Cell and Molecular Biology*.

[127] Camargo F. D., Gokhale S., Johnnidis J. B. (2007). YAP1 increases organ size and expands undifferentiated progenitor cells. *Current Biology*.

[128] Cao X., Pfaff S. L., Gage F. H. (2008). YAP regulates neural progenitor cell number via the TEA domain transcription factor. *Genes & Development*.

[129] Watt K. I., Judson R., Medlow P. (2010). Yap is a novel regulator of C2C12 myogenesis. *Biochemical and Biophysical Research Communications*.

[130] Lee J., Kim T., Yang T. (2008). A crucial role of WW45 in developing epithelial tissues in the mouse. *EMBO Journal*.

[131] Hong J.-H., Hwang E. S., McManus M. T. (2005). TAZ, a transcriptional modulator of mesenchymal stem cell differentiation. *Science*.

[132] Fernandez-L A., Northcott P. A., Dalton J. (2009). YAP1 is amplified and up-regulated in hedgehog-associated medulloblastomas and mediates Sonic hedgehog-driven neural precursor proliferation. *Genes & Development*.

[133] Alarcón C., Zaromytidou A.-I., Xi Q. (2009). Nuclear CDKs drive smad transcriptional activation and turnover in BMP and TGF-*β* pathways. *Cell*.

[134] Varelas X., Sakuma R., Samavarchi-Tehrani P. (2008). TAZ controls smad nucleocytoplasmic shuttling and regulates human embryonic stem-cell self-renewal. *Nature Cell Biology*.

[135] Varelas X., Miller B. W., Sopko R. (2010). The hippo pathway regulates Wnt/*β*-catenin signaling. *Developmental Cell*.

[136] Cao Q., Yu J., Dhanasekaran S. M. (2008). Repression of E-cadherin by the polycomb group protein EZH2 in cancer. *Oncogene*.

[137] Zhao X. D., Han X., Chew J. L. (2007). Whole-genome mapping of histone H3 Lys4 and 27 trimethylations reveals distinct genomic compartments in human embryonic stem cells. *Cell Stem Cell*.

[138] Pan G., Tian S., Nie J. (2007). Whole-genome analysis of histone H3 lysine 4 and lysine 27 methylation in human embryonic stem cells. *Cell Stem Cell*.

[139] Jiang W., Wang J., Zhang Y. (2013). Histone H3K27me3 demethylases KDM6A and KDM6B modulate definitive endoderm differentiation from human ESCs by regulating WNT signaling pathway. *Cell Research*.

[140] Winnier G., Blessing M., Labosky P. A., Hogan B. L. M. (1995). Bone morphogenetic protein-4 is required for mesoderm formation and patterning in the mouse. *Genes & Development*.

[141] Lewis S. L., Tam P. P. L. (2006). Definitive endoderm of the mouse embryo: formation, cell fates, and morphogenetic function. *Developmental Dynamics*.

[142] Borowiak M., Maehr R., Chen S. (2009). Small molecules efficiently direct endodermal differentiation of mouse and human embryonic stem cells. *Cell Stem Cell*.

[143] Wilson C. L., Matrisian L. M. (1996). Matrilysin: an epithelial matrix metalloproteinase with potentially novel functions. *The International Journal of Biochemistry & Cell Biology*.

[144] MacDougall J. R., Matrisian L. M. (1995). Contributions of tumor and stromal matrix metalloproteinases to tumor progression, invasion and metastasis. *Cancer and Metastasis Reviews*.

[145] Saarialho-Kere U. K., Crouch E. C., Parks W. C. (1995). Matrix metalloproteinase matrilysin is constitutively expressed in adult human exocrine epithelium. *Journal of Investigative Dermatology*.

[146] Fingleton B. M., Goss K. J. H., Crawford H. C., Matrisian L. M. (1999). Matrilysin in early stage intestinal tumorigenesis. *APMIS-Acta Pathologica, Microbiologica et Immunologica Scandinavica*.

[147] Crawford H. C., Matrisian L. M. (1995). Tumor and stromal expression of matrix metalloproteinases and their role in tumor progression. *Invasion and Metastasis*.

[148] McGuire J. K., Li Q., Parks W. C. (2003). Matrilysin (matrix metalloproteinase-7) mediates E-cadherin ectodomain shedding in injured lung epithelium. *The American Journal of Pathology*.

[149] Bair E. L., Massey C. P., Tran N. L. (2001). Integrin- and cadherin-mediated induction of the matrix metalloprotease matrilysin in cocultures of malignant oral squamous cell carcinoma cells and dermal fibroblasts. *Experimental Cell Research*.

[150] Davies G., Jiang W. G., Mason M. D. (2001). Matrilysin mediates extracellular cleavage of E-cadherin from prostate cancer cells: a key mechanism in hepatocyte growth factor/scatter factor-induced cell-cell dissociation and in vitro invasion. *Clinical Cancer Research*.

[151] Heppner K. J., Matrisian L. M., Jensen R. A., Rodgers W. H. (1996). Expression of most matrix metalloproteinase family members in breast cancer represents a tumor-induced host response. *The American Journal of Pathology*.

[152] Casey T., Bond J., Tighe S. (2009). Molecular signatures suggest a major role for stromal cells in development of invasive breast cancer. *Breast Cancer Research and Treatment*.

[153] Wheelock M. J., Buck C. A., Bechtol K. B., Damsky C. H. (1987). Soluble 80‐kd fragment of cell‐CAM 120/80 disrupts cell‐cell adhesion. *Journal of Cellular Biochemistry*.

[154] Noë V., Fingleton B., Jacobs K. (2001). Release of an invasion promoter E-cadherin fragment by matrilysin and stromelysin-1. *Journal of Cell Science*.

[155] Prehn R. T. (1994). Cancers beget mutations versus mutations beget cancers. *Cancer Research*.

[156] Jones J., Wang H., Zhou J. (2012). Nuclear kaiso indicates aggressive prostate cancers and promotes migration and invasiveness of prostate cancer cells. *The American Journal of Pathology*.

[157] Jones J., Wang H., Karanam B. (2014). Nuclear localization of Kaiso promotes the poorly differentiated phenotype and EMT in infiltrating ductal carcinomas. *Clinical & Experimental Metastasis*.

[158] Lozzio C. B., Lozzio B. B. (1975). Human chronic myelogenous leukemia cell-line with positive Philadelphia chromosome. *Blood*.

[159] Stone K. R., Mickey D. D., Wunderli H., Mickey G. H., Paulson D. F. (1978). Isolation of a human prostate carcinoma cell line (DU 145). *International Journal of Cancer*.

[160] Kaighn M. E., Narayan K. S., Ohnuki Y., Lechner J. F., Jones L. W. (1979). Establishment and characterization of a human prostatic carcinoma cell line (PC-3). *Investigative Urology*.

[161] Soule H. D., Vazguez J., Long A., Albert S., Brennan M. (1973). A human cell line from a pleural effusion derived from a breast carcinoma. *Journal of the National Cancer Institute*.

[162] Cailleau R., Olive M., Cruciger Q. V. J. (1978). Long-term human breast carcinoma cell lines of metastatic origin: preliminary characterization. *In Vitro Cellular & Developmental Biology - Animal*.

[163] Pan Y., Kytölä S., Farnebo F. (1999). Characterization of chromosomal abnormalities in prostate cancer cell lines by spectral karyotyping. *Cytogenetic and Genome Research*.

[164] van Bokhoven A., Varella-Garcia M., Korch C. (2003). Molecular characterization of human prostate carcinoma cell lines. *The Prostate*.

[165] Xu J., Chambers A. F., Tuck A. B., Rodenhiser D. I. (2008). Molecular cytogenetic characterization of human breast cancer cell line MDA-MB-468 and its variant 468LN, which displays aggressive lymphatic metastasis. *Cancer Genetics and Cytogenetics*.

[166] Gribble S. M., Roberts I., Grace C., Andrews K. M., Green A. R., Nacheva E. P. (2000). Cytogenetics of the chronic myeloid leukemia-derived cell line K562 karyotype clarification by multicolor fluorescence in situ hybridization, comparative genomic hybridization, and locus-specific fluorescence in situ hybridization. *Cancer Genetics and Cytogenetics*.

[167] Cowan C. A., Klimanskaya I., McMahon J. (2004). Derivation of embryonic stem-cell lines from human blastocysts. *The New England Journal of Medicine*.

[168] Maitra A., Arking D. E., Shivapurkar N. (2005). Genomic alterations in cultured human embryonic stem cells. *Nature Genetics*.

[169] Catalina P., Montes R., Ligero G. (2008). Human ESCs predisposition to karyotypic instability: is a matter of culture adaptation or differential vulnerability among hESC lines due to inherent properties?. *Molecular Cancer*.

[170] Lefort N., Feyeux M., Bas C. (2008). Human embryonic stem cells reveal recurrent genomic instability at 20q11.21. *Nature Biotechnology*.

[171] Spits C., Mateizel I., Geens M. (2008). Recurrent chromosomal abnormalities in human embryonic stem cells. *Nature Biotechnology*.

[172] Thomson J. A., Itskovitz-Eldor J., Shapiro S. S. (1998). Embryonic stem cell lines derived from human blastocysts. *Science*.

[173] Hanahan D., Weinberg R. A. (2000). The hallmarks of cancer. *Cell*.

[174] Storchova Z., Pellman D. (2004). From polyploidy to aneuploidy, genome instability and cancer. *Nature Reviews Molecular Cell Biology*.

[175] Toyota M., Ohe-Toyota M., Ahuja N., Issa J.-P. J. (2000). Distinct genetic profiles in colorectal tumors with or without the CpG island methylator phenotype. *Proceedings of the National Acadamy of Sciences of the United States of America*.

[176] Baylin S. B., Herman J. G. (2000). DNA hypermethylation in tumorigenesis: epigenetics joins genetics. *Trends in Genetics*.

[177] Lengauer C., Kinzler K. W., Vogelstein B. (1998). Genetic instabilities in human cancers. *Nature*.

[178] Kahan B. W., Ephrussi B. (1970). Developmental potentialities of clonal in vitro cultures of mouse testicular teratoma. *Journal of the National Cancer Institute*.

[179] Evans M. J. (1972). The isolation and properties of a clonal tissue culture strain of pluripotent mouse teratoma cells. *Journal of Embryology and Experimental Morphology (JEEM)*.

[180] Lehman J. M., Speers W. C., Swartzendruber D. E., Pierce G. B. (1974). Neoplastic differentiation: Characteristics of cell lines derived from a murine teratocarcinoma. *Journal of Cellular Physiology*.

[181] Mintz B., Illmensee K. (1975). Normal genetically mosaic mice produced from malignant teratocarcinoma cells. *Proceedings of the National Acadamy of Sciences of the United States of America*.

[182] Illmensee K., Mintz B. (1976). Totipotency and normal differentiation of single teratocarcinoma cells cloned by injection into blastocysts. *Proceedings of the National Acadamy of Sciences of the United States of America*.

[183] Mccullough K. D., Coleman W. B., Ricketts S. L., Wilson J. W., Smith G. J., Grisham J. W. (1998). Plasticity of the neoplastic phenotype in vivo is regulated by epigenetic factors. *Proceedings of the National Acadamy of Sciences of the United States of America*.

[184] Gerschenson M., Graves K., Carson S. D., Wells R. S., Pierce G. B. (1986). Regulation of melanoma by the embryonic skin. *Proceedings of the National Acadamy of Sciences of the United States of America*.

[185] Holm S. (2002). Going to the roots of the stem cell controversy. *Bioethics*.

[186] De Wert G., Berghmans R. L., Boer G. J. (2002). Ethical guidance on human embryonic and fetal tissue transplantation: a European overview. *Medicine, Health Care and Philosophy*.

[187] De Wert G., Mummery C. (2003). Human embryonic stem cells: research, ethics and policy. *Human Reproduction*.

[188] Lo B., Parham L. (2009). Ethical issues in stem cell research. *Endocrine Reviews*.

[189] Odom D. T., Dowell R. D., Jacobsen E. S. (2007). Tissue-specific transcriptional regulation has diverged significantly between human and mouse. *Nature Genetics*.

[190] Zheng W., Gianoulis T. A., Karczewski K. J., Zhao H., Snyder M. (2011). Regulatory variation within and between species. *Annual Review of Genomics and Human Genetics*.

[191] Yue F., Cheng Y., Breschi A. (2014). A comparative encyclopedia of DNA elements in the mouse genome. *Nature*.

[192] Denas O., Sandstrom R., Cheng Y. (2015). Genome-wide comparative analysis reveals human-mouse regulatory landscape and evolution. *BMC Genomics*.

[193] Pierce G. B. (1967). Teratocarcinoma: model for a developmental concept of cancer. *Current Topics in Developmental Biology*.

[194] Markert C. L. (1968). Neoplasia: a disease of cell differentiation. *Cancer Research*.

[195] Pierce G. B., Johnson L. D. (1971). Differentiation and cancer. *In Vitro: Journal of the Tissue Culture Association*.

[196] Needham J. (1936). New advances in the chemistry and biology of organized growth:(section of pathology). *Proceedings of the Royal Society of Medicine*.

[197] Smithers D. W. (1962). Cancer an attack on cytologism. *The Lancet*.

[198] Soto A. M., Sonnenschein C. (2005). Emergentism as a default: cancer as a problem of tissue organization. *Journal of Biosciences*.

